# Mean annual temperature influences local fine root proliferation and arbuscular mycorrhizal colonization in a tropical wet forest

**DOI:** 10.1002/ece3.6561

**Published:** 2020-08-28

**Authors:** Suzanne Pierre, Creighton M. Litton, Christian P Giardina, Jed P. Sparks, Timothy J. Fahey

**Affiliations:** ^1^ Department of Ecology and Evolutionary Biology Cornell University Ithaca New York USA; ^2^ Department of Integrative Biology University of California, Berkeley Berkeley California USA; ^3^ Department of Natural Resources and Environmental Management University of Hawai'i at Manoa Honolulu Hawai'i USA; ^4^ Institute of Pacific Islands Forestry Pacific Southwest Research Station US Forest Service Hilo Hawaii USA; ^5^ Department of Natural Resources Cornell University Ithaca New York USA

**Keywords:** arbuscular mycorrhizal fungi, fine roots, mean annual temperature, nitrogen availability, root proliferation, tropical montane wet forest

## Abstract

Mean annual temperature (MAT) is an influential climate factor affecting the bioavailability of growth‐limiting nutrients nitrogen (N) and phosphorus (P). In tropical montane wet forests, warmer MAT drives higher N bioavailability, while patterns of P availability are inconsistent across MAT. Two important nutrient acquisition strategies, fine root proliferation into bulk soil and root association with arbuscular mycorrhizal fungi, are dependent on C availability to the plant via primary production. The case study presented here tests whether variation in bulk soil N bioavailability across a tropical montane wet forest elevation gradient (5.2°C MAT range) influences (a) morphology fine root proliferation into soil patches with elevated N, P, and N+P relative to background soil and (b) arbuscular mycorrhizal fungal (AMF) colonization of fine roots in patches. We created a fully factorial fertilized root ingrowth core design (N, P, N+P, unfertilized control) representing soil patches with elevated N and P bioavailability relative to background bulk soil. Our results show that percent AMF colonization of roots increased with MAT (*r* ^2^ = .19, *p* = .004), but did not respond to fertilization treatments. Fine root length (FRL), a proxy for root foraging, increased with MAT in N+P‐fertilized patches only (*p* = .02), while other fine root morphological parameters did not respond to the gradient or fertilized patches. We conclude that in N‐rich, fine root elongation into areas with elevated N and P declines while AMF abundance increases with MAT. These results indicate a tradeoff between P acquisition strategies occurring with changing N bioavailability, which may be influenced by higher C availability with warmer MAT.

## Graphical abstract

Rising mean annual temperature (MAT) is predicted to increase the availability of inorganic nitrogen (IN) for biological use via impacts on rates of biogeochemical cycling, and with implications for plant N and phosphorus (P) acquisition. We found that fine root proliferation into N+P‐fertilized microsites declined across a natural temperature and soil‐IN gradient, while fine root mycorrhizal colonization increased across the gradient, with no effect of fertilization treatment. We conclude that in this tropical montane forest, plants may prioritize a mycorrhizal nutrient acquisition strategy over fine root proliferation in high MAT/N bioavailability conditions as a compensatory P acquisition strategy.

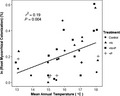

## 1 INTRODUCTION

Increasing air temperature resulting from greenhouse gas forcing is expected to affect terrestrial primary production (Luyssaert et al., 2007) and alter aboveground and belowground plant carbon (C) allocation (Litton & Giardina, 2008; Raich & Nadelhoffer, 1989). These temperature‐driven changes in C fixation and allocation may induce feedbacks that alter the carbon dioxide (CO_2_) balance of forest ecosystems (Vogel et al., 2008). Belowground C allocation comprises a significant and variable proportion of gross primary production (GPP) (Litton, Raich, & Ryan, 2007) supporting various belowground plant processes. The amount of C fixed during photosynthesis and the proportion of biomass C allocated to distinct belowground pools (*e.g.,* fine roots, mycorrhizae) are ultimately important for the formation and decomposition rates of soil organic matter (SOM), in turn influence the rate of CO_2_ efflux to the atmosphere (Kuzyakov & Schneckenberger, 2004; Schmidt et al., 2011).

The bioavailability of soil nutrients, in particular nitrogen (N) and phosphorus (P), has been tied to the allocation of C to root structures and symbioses (mycorrhizas, root nodules for N‐fixation) that promote belowground nutrient acquisition (BassiriRad, 2000; Melillo et al., 2011; Pendall et al. 2004; Reich et al., 2014). Within the conceptual framework of nutrient acquisition strategy, plants adjust the proportion of net primary production (NPP) allocated to belowground components in order to overcome growth limitation (Bloom, Chapin, & Mooney, 1985; Treseder & Vitousek, 2001). As a result, we observe globally distributed patterns in nutrient acquisition strategies related to soil age and disturbance history (Lambers, Raven, Shaver, & Smith, 2008). While the relationship between lithology and soil nutrient acquisition is an important topic in ecosystems ecology (Walker & Syers, 1976), exactly how global climate changes, such as rising mean annual temperature (MAT), influence soil resource availability and plant nutrient acquisition under natural conditions remains poorly understood (Gill & Jackson, 2000).

Natural field observations (Ostertag, 2001), field fertilization experiments (Haynes & Gower 1995), and modeling exercises (Dybzinski, Farrior, Wolf, Reich, & Pacala, 2011) have all shown that increasing soil nutrient availability in forest ecosystems leads to reduced belowground C allocation and proportional increases in aboveground C allocation (Litton et al., 2007). Fertilization studies show that plants in nutrient‐poor soils allocate a greater proportion of total plant C to fine roots, root exudates, and fungal symbionts, compared to those in nutrient‐rich sites (Johnson et al. 2010; Treseder & Vitousek, 2001; Vicca et al., 2012). Phosphorus limits productivity in highly weathered soils typical of the global tropics and in volcanic soils where P availability is limited by biological and mineral occlusion (Olander & Vitousek, 2005), while N limits forest production in northern latitudes where younger, recently glaciated soils dominate (LeBauer & Treseder, 2008; Reich & Oleksyn, 2004). Fine root biomass (FRB) and fine root length (FRL) determine the root surface area available for soil exploration and nutrient uptake, and so provide indices of plant demand for nutrients (Berntson, Farnsworth, & Bazzaz, 1995; Powers et al., 2004). Fine root proliferation into nutrient‐enriched areas of bulk soil, described as “patches”, also depends on the ability of roots to elongate and proliferate in response to patchy distribution of soil resources (Adams, McCormack, & Eissenstat, 2013; Farley & Fitter, 1999).

Plant association with the major types of mycorrhizal fungi, variations in root/mycorrhizal morphology (Chen et al., 2016), and nutrient translocation within the root (Marschner & Dell, 1994) are also critical to the nutrition of terrestrial plants (Averill, Bhatnagar, Dietze, Pearse, & Kivlin, 2019). Arbuscular mycorrhizal fungi (AMF) support plant nutrient acquisition, particularly of P, via uptake by extraradical hyphae and translocation of nutrients in exchange for plant C within arbuscules and intracellular hyphal coils (Smith & Smith, 2011). Across tree species, nutrient foraging by AMF, inferred from extraradical hyphal length and biomass (mycelium) in nutrient patches, is less precise compared to foraging precision in ectomycorrhizal fungi, across AMF‐associated tree species (Chen et al., 2016; Cheng et al., 2016), while the presence of intraradical AMF structures, an index of P translocation, is related to overall benefit to plant biomass across ecosystems (Treseder, 2013). Therefore, abundance of fungal structures in roots may indicate overall plant dependence on AMF more directly than extraradical AMF hyphae when not differentiating among AMF species (Chen et al., 2016; Hart & Reader, 2002). Plant colonization by AMF is more prevalent under conditions of nutrient limitation of plant growth, particularly in P‐limited environments (Johnson, Wolf, & Koch, 2003). In soils with high N:P, root colonization by AMF, frequently quantified as percent root length colonized (Vierheilig et al., 2005), is increasingly advantageous and competition among mycorrhizal and nonmycorrhizal fungi for plant C favors AMF (Chagnon & Bradley, 2013; Johnson, Graham, & Smith, 1997; Johnson et al. 2010). These patterns illustrate the link between biogeochemical processes mediated by free‐living soil microorganisms and the prevalence of the root/AMF symbiosis via soil N:P (Okiobe et al., 2019; Veresoglou et al., 2019). In addition to soil nutrient conditions, exchange of nutrients for C between AMF and plants is influenced by the availability of C in live biomass (Brzostek, Fisher, & Phillips, 2014; Peng et al., 1993). Temperature has been shown to directly limit the transfer of P from AMF to plants, potentially via effects on photosynthesis (Gavito et al. 2003; Hammer, Pallon, Wallander, & Olsson, 2011; Olsson et al., 2010). Therefore, temperature influences more than one of the ecological conditions which drive the extent of AMF colonization (Heinemeyer & Fitter, 2004), and stand‐scale tests of the relationship between rising temperature, fine root proliferation, and AMF abundance are necessary to characterize the effects of climate warming on soil C and nutrient economies.

Natural elevation gradients provide an ideal setting to test the role of environmental variation in ecological processes in ways that more accurately represent real ecosystems than manipulation studies that often produce transient effects and experimental artifacts (Fukami & Wardle, 2005; Malhi et al., 2010; Sundqvist et al., 2013). Giardina, Litton, Crow, and Asner (2014) used a natural but highly constrained elevation gradient on the island of Hawaii to show that MAT is positively related to the total flux of autotrophic C belowground, as well as soil CO_2_ efflux (Litton, Giardina, Albano, Long, & Asner, 2011). Across the same tropical montane wet forest elevation gradient as in Giardina et al. (2014), we previously showed that soil N bioavailability increases with MAT, which suggests that increasing C fluxes and N bioavailability may be linked to warming MAT through soil microbial mechanisms (Pierre et al., 2017). Previous research has suggested that increases in N cycling and availability with warming could contribute to higher forest productivity with climate change (Cleveland et al., 2011; LeBauer & Treseder, 2008; Rustad et al., 2001).

In the present study, we leveraged the permanent plots located along a MAT/elevation gradient on Hawaii to investigate the effects of MAT and local soil nutrient bioavailability on fine root proliferation within nutrient‐fertilized patches across a 5.2°C range of MAT (Litton et al., 2011). Ecological variables other than temperature (soil moisture, plant community composition, successional stage, soil type, lithology) are constant across temperature gradient (Giardina et al., 2014; Litton et al., 2011; Selmants et al., 2014). We determined how fine root proliferation into N‐ and P‐fertilized patches (defined here as ~5.25 cm^3^ of soil) respond to increasing MAT and site fertility in situ. We hypothesized that the increasing magnitude of ecosystem C fluxes with MAT would drive an overall increase in fine root proliferation and root mycorrhizal colonization at the plot level, while fine root proliferation into N‐fertilized patches would with declining native soil N bioavailability. Conversely, we hypothesized that fine root proliferation within P‐fertilized patches would increase with MAT and soil N bioavailability, as increasing soil N bioavailability with warming would increase soil N:P and drive greater P demand (Hendricks, Nadelhoffer, & Aber, 1993). We also determined the abundance of AMF within ingrowth fine roots as % root length colonized, an indication of plant community P status (Treseder, 2013), and anticipated that all ingrowth roots across treatments would reflect a shift toward P acquisition via increased abundance of AMF structures with increasing MAT and soil N bioavailability. Higher root colonization by AMF with increasing MAT would indicate a compensatory mechanism for plant P acquisition in response to increasing bioavailability of mineral N with warmer MAT (Pierre et al., 2017).

## 2 METHODS

### 2.1 Study site

This study was conducted along an 800 m elevation gradient on the northeastern slope of the Mauna Kea Volcano on the Island of Hawaii (Litton et al., 2011). Nine permanent research plots (20 × 20 m) located in native‐dominant, mature tropical montane wet forest were included this study (Table 1). The seven lower‐elevation permanent plots are located in the Hawai'i Experimental Tropical Forest (HETF; 19°56′41.3″N, 155°15′44.2″W; 600–1,800 m.a.s.l) and the two higher‐elevation plots in the adjacent Hakalau Forest National Wildlife Refuge (HFNWR; 19°50′31.3″N, 155°17′35.2″W; 600–2,000 m.a.s.l). All plots are characterized as *Metrosideros polymorpha* Gaudich. –*Acacia koa* A. Gray forests. *M. polymorpha* and *Cheirodendron trigynum* (Gaudich.) A. Heller dominate the canopy and midstory, respectively, across all plots (84%–97% of basal area excluding tree ferns). Additionally, three species of tree ferns (*Cibotium* spp.; midstory) make up an average 46% of stand basal area in these plots (Litton et al., 2011). Arbuscular mycorrhizal fungi associate with >90% of plant species endemic to the Hawaiian islands and are therefore the focal mycorrhizal type in this study (Koske, Gemma, & Flynn, 1992).

**TABLE 1:** Climate and stand data for 9 permanent plots located in a tropical wet montane forest on Mauna Kea, Hawaii, USA

Elevation (m.a.s.l.)	Plot name	MAT (°C)^b^	MAP (mm)^a^	Soil temperature (°C)^b^	Soil VWC (%)^b^	Total stand BA (m^2^/ha)^b^	Stand density (individuals/ha)^b^
800	SPE800	18.2	4,204	18.15	53.81	116	4,225
934	SPE934	17.3	4,133	16.98	55.84	100	3,300
1,024	SPE1024	16.7	4,043	16.33	54.19	97	3,750
1,116	SPE1116	16.1	3,988	15.80	51.19	155	4,275
1,116	WPL1116	16.1	3,714	15.20	33.94	109	5,875
1,204	WPL1204	15.5	3,521	15.59	30.66	102	3,900
1,274	WPL1274	15.1	3,448	15.01	32.06	81	4,375
1,468	HKL1468	13.8	3,488	14.09	50.66	54	13,200
1,600	HKL1600	13.00	3,195	13.18	53.59	66	16,400

Note

Plots comprise a 5.2°C mean annual temperature (MAT) gradient.

^a^ Mean annual precipitation (MAP) data from Giambelluca et al. (1986).

^b^ Data from Litton et al. (2011).

Plots along the elevation gradient vary in MAT from 13°C at the highest elevation plot (1,600 m.a.s.l.) to 18.2°C at the lowest elevation plot (800 m.a.s.l.), thus forming a 5.2°C MAT gradient (Table 1). Soil water balance is relatively constant across all plots due to a concomitant decline in mean annual precipitation with increasing elevation (Litton et al., 2011; Selmants et al., 2014). Substrate in all plots is derived from ~20 ky (14–65 ky) weathered tephra, and soils are moderate to well‐drained hydrous, ferrihydritic/amorphic, isothermic/isomesic Acrudoxic Hydrudands of the Akaka, Honokaa, Maile, and Piihonua soil series (Soil Survey Staff 2010). Mean soil pH is 3.9 and base saturation and estimated mean cation exchange capacity are 32.4% and 11.9 cmol/kg, respectively (Litton et al., 2011). Mean soil (0–10 cm) bulk density and carbon content across the MAT gradient are 0.21 g/cm^3^ and 14%, respectively. Soil nitrate (${\text{NO}}_{3}^{‐}$) bioavailability in these plots, measured using ion exchange resins (Western Ag, Saskatoon, SK, Canada), increased linearly with MAT (*r* ^2^ = .79, *p* = .003; Pierre et al., 2017).

### 2.2 Fertilized root ingrowth core construction and placement

Cores were constructed of polyvinyl mesh netting with 1 mm × 1.5 mm holes (open area = 50%), 10 cm in height and 7.5 cm in diameter, and sewn closed using nylon thread. Each core was filled with ~25 g (~5.25 cm^3^) of a mixture of a calcined clay pellets (Turface®) and vermiculite matching the average bulk density of 0.21 g/cm^3^ of native soils across the gradient. This mixture was selected for the absence of background N and P in calcined clay, and the modulation of bulk density by the vermiculite fraction (Raich et al., 1994). For fertilization, we approximated the fertilization rate for N and P (40 g/m^2^) achieved by previous root ingrowth core studies in very similar tropical montane wet forests on the island of Hawaii (Raich et al., 1994). Cores received a one‐time dose of 100 ml of deionized water (control), 6.016 g/L urea (CH_4_N_2_O) (N treatment), 21.69 g/L Na_3_PO_4_·H_2_O (P treatment), or a combined solution of both solutes at the same concentrations (N+P treatment) Riley and Vitousek 1995. Each treatment was applied with a needle and syringe by evenly injecting and releasing small aliquots totaling 100 ml throughout the media (Raich et al., 1994). Cores were covered and air‐dried at laboratory temperature overnight, and then weighed and stored in plastic bags at room temperature until burial. Based on previous studies, fertilization treatments were assumed to be largely retained by the calcined clay media within the cores across the study period (Raich et al., 1994). Because a standard amount of each fertilization treatment was added to cores, the contrast between naturally available soil N and N added in ingrowth cores varied across the gradient with the natural change in bulk N (Table 2), but differences in N contrast among plots were assumed to be negligible compared to the difference in N availability between the ingrowth core and the background soil.

**TABLE 2:** Soil characteristics for 9 permanent plots located in tropical montane wet forest on Mauna Kea, Hawaii, USA

Plot elevation (m.a.s.l.)	Soil series^a^	Soil pH^b^	Bulk density (g/cm^3^)	Soil C stock (0–10 cm; g C m^−2^)^c^	Soil N stock (0–10 cm; g N m^−2^)^d^	Soil bioavailable ${\text{NO}}_{3}^{‐}$ (mg‐N m^−2^ d^−1^)^d^	Soil bioavailable ${\text{NH}}_{4}^{+}$ (mg‐N m^−2^ d^−1^)^d^
800	Akaka	4.1	0.21	4,066.7	162.6	2.159	0.1073
934	Akaka	4.2	0.19	4,400.1	291.2	0.816	0.1277
1,024	Akaka	3.7	0.19	2,562.4	152.5	1.234	0.07711
1,116	Akaka	3.8	0.20	2,662.8	157.3	1.389	0.5905
1,116	Honokaa	3.6	0.26	‐	‐	‐	‐
1,204	Honokaa	3.7	0.23	4,139.4	192.1	0.6003	0.1986
1,274	Maile	3.9	0.22	3,674.2	29.0	0.6766	0.7173
1,468	Akaka	4.2	0.18	2,958.3	151.6	0.01587	0.06333
1,600	Piihonua	4.1	0.23	5,666.0	311.9	0.05214	0.5883

Note

Plots comprise a 5.2°C mean annual temperature (MAT) gradient.

^a^ Soils in all plots are classified as hydrous, ferrihydritic/amorphic, isothermic/isomesic Acrudoxic Hydrudands (Litton et al., 2011).

^b^ Quantified on fresh soils (*n* = 10 soil cores plot^−1^ to 10 cm depth) (Litton et al., 2011).

^c^ From Litton et al. (2011).

^d^ From Pierre et al. (2017).

Between August 25–29, 2015, replicates of each treatment (Control, +N, +P, N+P) were buried to 10 cm depth in each of the nine plots in clusters spaced ~30 cm apart. Five replicate clusters of fertilized cores were buried per plot (total of 5 treatment replicates within 9 permanent plots; *n* = 180). Replicate clusters were placed at the corners of each 20 × 20 m plot, with the fifth cluster placed equidistant between two of the corners on the perimeter of the plot. All cores were collected between November 1–11, 2015, for an average burial time of 75 days. Three cores were lost in the field, for a total of *n* = 177 cores returned to the laboratory.

Cores were stored in plastic zip‐lock bags and placed in insulated containers for immediate transport to the laboratory, where they were stored at ~1.5°C until root sampling approximately 10 days later. Fine roots (<2 mm) were removed from the media using a fine (<1 mm) sieve and forceps. After rinsing with deionized water, the fresh fine root sample was weighed for fine root biomass. The average length of a single intact (*i.e.,* not damaged or fragmented) root found inside an ingrowth core was ~5 ± 1 cm, and this length was chosen as the standard subsample length to allow for comparison of AMF colonization between plots. A subsample of ~5 ± 1 cm of root length was taken from the total fresh root sample in each ingrowth core to store in 50% ethanol until clearing and staining for mycorrhizal colonization (Grace & Stribley, 1991). The remaining fine roots in each sample were weighed, dried at 60°C for 72 hr, and reweighed to calculate moisture content (Figure S1). The subsamples used for quantifying mycorrhizal colonization were similarly weighed and dried following visual quantification 200× magnification under microscope. The total dried root sample was then scanned using a digital scanner, and FRL was measured using the WinRHIZO software (Régent Inc.), and specific root length (SRL = FRB/FRL; g/cm) was determined from these measurements Ostonen et al.^2007^. Drying roots prior to scanning is a methodological limitation of this study, as drying may have affected root length and because fine roots of plant species within plots may have different drying effects. For our analyses, we assume equivalent laboratory drying effects on fine roots of species within and between plots similarly.

### 2.3 Mycorrhizal colonization

The extent of root colonization by AMF was estimated using the percent root length colonization method (Biermann & Linderman, 1981; McGonigle et al., 1990; Toth et al., 1991). This is the standard method of quantifying AMF abundance within plants (Vierheilig et al., 2005) through standardized observations of the proportion of root length containing intraradical AMF structures (arbuscules, intracellular hyphae, and vesicles) (Biermann & Linderman, 1981; Treseder, 2013). From the total ingrowth cores collected from the field (*n* = 177) collected from the field, a subset contained ≥5 cm total fine root length (*n* = 108) and a further subset (*n = *37) contained ≥5 cm of fine roots that could be chemically cleared in a reasonable amount of time (<24 hr) for quantifying intraradical AMF structures. Fine root subsamples for mycorrhizal colonization were cleared and stained with vinegar and ink following the methods of Brundrett and Abbott (1994), reviewed by Vierheilig et al. (2005). Briefly, roots were cleared in 10% KOH solution and autoclaved on a liquid cycle in 10‐min intervals until roots were cleared of all pigment (maximum 24 hr), with KOH solution changed between each autoclave cycle. Roots were then stained in 0.05% Parker Quink black ink (Parking Pen Products) in a 1:1 solution of glycerol and vinegar for ~24 hr. Roots were cut with scissors into 2 cm segments and mounted on glass slides parallel to one another. Roots were observed at 200× magnification, with two lateral visual scans from one end of the microscope slide to the other across the upper and lower halves of the slide. Five equally spaced lines were marked on the slide demarking points for observation, which produced a total of 10 observation points per slide. When a point was met in a visual scan, an observation was made for the presence or absence of fungal structures (arbuscules and intercellular hyphae). Total root mycorrhizal colonization was then quantified as the percentage of observation points where mycorrhizal structures were present. Observations were made by three different individuals to minimize observer bias, and percent colonization is an average of three values per sample. A total of 37 root samples were scored, the remainder being too small to make these measurements.

### 2.4 Statistical analysis

Fine root length, FRB, SRL, and AMF abundance were determined to be non‐normally distributed by a Shapiro–Wilk test and were log transformed to meet assumptions of normality for subsequent statistical tests. The responses of these root variables to MAT and natural soil ${\text{NO}}_{3}^{‐}$ bioavailability were then explored by testing linear models of for additive and interactive effects, and significant responses were determined using *t* tests. Third, linear mixed effects (LME) regression models were used to determine the significance of individual and interacting fixed (*i.e.,* observed or manipulated) variables while accounting for random (*i.e.,* uncontrolled or unobserved) effects in this study. Fixed effects in the LME model were MAT (Table 1), the natural soil ${\text{NO}}_{3}^{‐}$ bioavailability (Table 2), and fertilization treatment. Random effects included in the LME were the plot and ingrowth core clusters within plots. Reduced and full (*i.e.,* including observed and treatment variables) LME models were compared by Akaike's information criterion (AIC). Estimated marginal means (EM means) (*i.e.,* frequency‐adjusted), rather than arithmetic means, were then determined from the selected LME model, to adjust for unbalanced quantities of root ingrowth cores collected from plots, as some ingrowth cores were lost in the field (Harrison et al., 2018; Searle et al., 1980). Estimated marginal means were also calculated in order to determine the significance of the three‐way interaction of MAT, soil ${\text{NO}}_{3}^{‐}$ bioavailability, and nutrient patch availability. Post hoc contrasts of fine root responses to fertilization treatments at every observed value of MAT and soil ${\text{NO}}_{3}^{‐}$ bioavailability were conducted by ANOVA using Tukey's method and a 95% confidence interval. The significant interaction effects on the EM means derived from the selected LME model were visualized as interaction plots using the emmip function in the emmeans package ( R Core Team, 2016). The LME model estimates visualized in the interaction plot were simplified by specifying only three observed levels of ${\text{NO}}_{3}^{‐}$ bioavailability (Low = 0.25 mg N m^−2^, Mid = 0.75 mg N m^−2^, High = 1.25 mg N m^−2^) in the LME model, rather than plotting all 9 observed values of soil ${\text{NO}}_{3}^{‐}$ bioavailability (see Table 1). Finally, percent root length colonized by AMF was measured for 37 ingrowth cores, which had unbalance sample sizes among treatments and plots. Response of AMF root colonization to MAT, ${\text{NO}}_{3}^{‐}$ bioavailability, and fertilization treatment was determined through ANOVA and a post hoc Tukey's honest significant difference test to account for unequal samples between plots and treatments in the AMF colonization subsample. All statistical analyses were performed in R (R Core Team, 2016).

## 3 RESULTS

**FIGURE 1:**
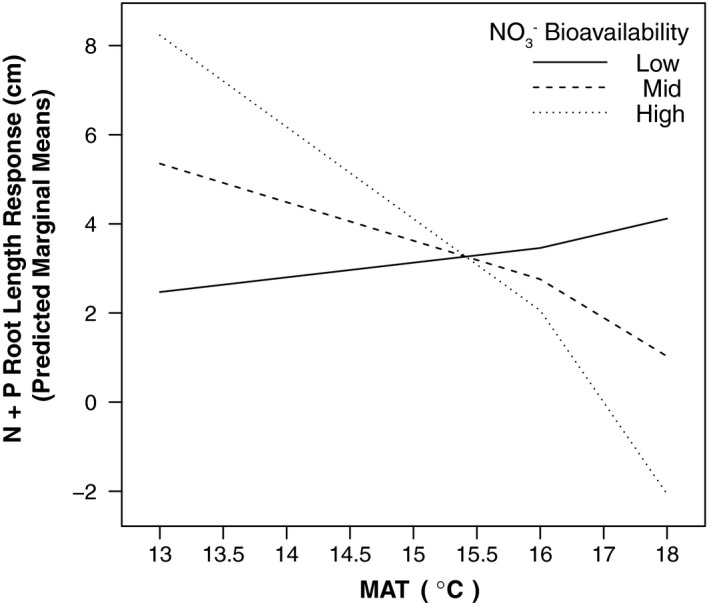
A plot of estimated marginal mean length (cm; log scale) of fine root ingrowth within nitrogen and phosphorus (N+P)‐fertilized root ingrowth cores across a MAT gradient (*x* axis) at three observed levels of NO₃⁻ bioavailability (lines; Low = 0.25 mg N m^−2^, Mid = 0.75 mg N m^−2^, High = 1.25 mg N m^−2^). Measurements were made in 9 permanent plots located in tropical montane wet forest on Mauna Kea, Hawaii, USA. Plots comprise a 5.2°C mean annual temperature (MAT) gradient. The *y*‐axis shows the difference in FRL from a null response, for which FRL response equals zero, predicted by a linear mixed effects regression model. The root length response to the N+P treatment was significantly different from other treatments (*p* = .023) and the root length response to N+P at High, Mid, and Low ${\text{NO}}_{3}^{‐}$ availability significantly responded to MAT (*p = *.02)

Data and statistical models show MAT (*p* = .041), natural soil ${\text{NO}}_{3}^{‐}$ bioavailability (*p = *.019), (from here on referred to as “bioavailable ${\text{NO}}_{3}^{‐}$”), and their interaction (*p* = .017) positively correlate with FRL in ingrowth cores (Table 2, Model 2). Inclusion of fertilization treatments in the linear mixed effects model showed a negative trend for FRL response to fertilized ingrowth cores with increasing MAT and soil bioavailable ${\text{NO}}_{3}^{‐}$ (Table 3, Model 1). This decline in FRL across MAT was only significant for N+‐P fertilized cores (*p = *.02; Figure 1). Fine root biomass and SRL showed no response to the MAT gradient, ${\text{NO}}_{3}^{‐}$ bioavailability, or fertilization. Fine root moisture was invariant across MAT (Figure S1). These results suggest the direction of the FRL response to MAT and ${\text{NO}}_{3}^{‐}$ bioavailability is sensitive to the combination of elevated N and P in patches. Figure 1 visualizes the FRL response to N+P fertilization as an estimated marginal means interaction plot for Model 1 (Table 3), showing that the interaction between MAT and ${\text{NO}}_{3}^{‐}$ bioavailability significantly influences the slope of the FRL response to the N+P treatment.

**TABLE 3:** Three linear mixed effects models comparing inclusion of observed variables only (natural soil nitrate (${\text{NO}}_{3}^{‐}$) bioavailability, mean annual temperature (MAT); Model 2, Model 3) and including both observed and manipulated variables (phosphorus (+P) and nitrogen (+N), and combined nitrogen (N) and P (N+P) root ingrowth core fertilization; Model 1) for estimating fine root ingrowth length (cm)

	Model 1	Model 2	Model 3
MAT	0.057 (0.359)	0.558** (0.264)	−0.039 (0.192)
Bioavailable nitrate	10.425 (13.270)	23.619** (9.756)	
+N	−6.443 (5.729)		
+N+P	−13.663*** (5.838)		
+P	−10.794* (5.716)		
MAT:Bioavailable nitrate	−0.706 (0.856)	−1.567** (0.630)	
AIC	735.631	732.344	739.474

Note

Random effects included in the models (not shown) include within‐plot ingrowth core placement and plot effect not accounted for by experimental design. Measurements were made in 9 permanent plots located in tropical montane wet forest on Mauna Kea, Hawaii, USA. Plots comprise a 5.2°C mean annual temperature (MAT) gradient.

*** *p *< .01.

** *p *< .05.

* *p *< .1.

**FIGURE 2:**
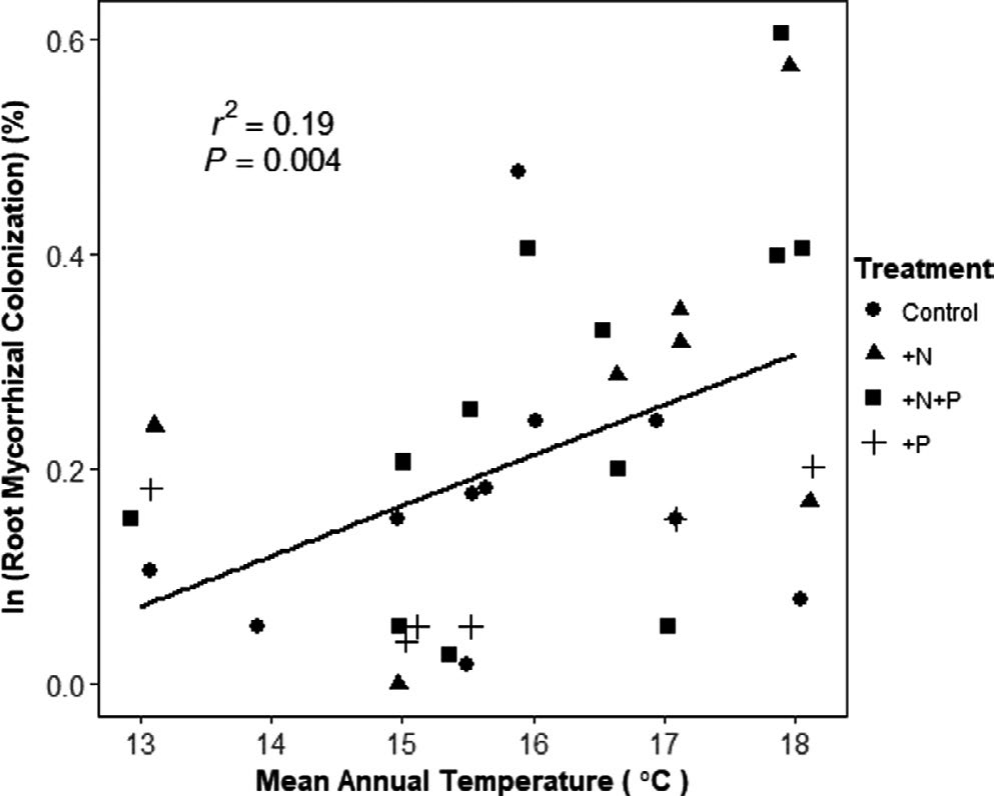
Arbuscular mycorrhizal fungal (AMF) colonization (% of root length colonized, log scale) of fine roots in fertilized root ingrowth cores measured in 9 permanent plots located in tropical montane wet forest on Mauna Kea, Hawaii, USA. Plots comprise a 5.2°C mean annual temperature (MAT) gradient. AMF colonization increases linearly with mean annual temperature (MAT) (*r* ^2^ = .43; *p* = .0039). Each point represents % mycorrhizal colonization of roots subsampled from a core. Mycorrhizal colonization did not differ among fertilization treatments

Arbuscules and intercellular hyphae were observed in fine roots collected from fertilized root ingrowth cores. Arbuscular mycorrhizal colonization of fine roots increased linearly with MAT (*p = *.0022, Figure 2), with a mean increase of 0.074% (SE ± 0.022) per 1°C increase in MAT. Fine root AMF colonization did not respond to ${\text{NO}}_{3}^{‐}$ bioavailability (*p* = .25). No fertilization treatments influenced the percent mycorrhizal colonization observed in fine root ingrowth, and no significant difference was shown for between fertilization treatments (*p* > .05).

## 4 DISCUSSION

### 4.1 Fine root responses to nutrient availability and MAT

Fine root proliferation into fertilized patches responded positively to warming (MAT) and the concomitant increases soil ${\text{NO}}_{3}^{‐}$ bioavailability along this tropical montane wet forest MAT gradient. As these environmental drivers of fine root proliferation (discussed here in terms of FRL) increase, they also show a statistical interaction that specifically reduces fine root proliferation into soil patches (ingrowth cores) with elevated N and P concentrations. These divergent results suggest two distinct influences of MAT and ${\text{NO}}_{3}^{‐}$ bioavailability on fine root proliferation into nutrient‐enriched patches. First, increasing temperature and resultant increases in bulk ${\text{NO}}_{3}^{‐}$ bioavailability (Pierre et al., 2017) appear to drive increasing total belowground C flux across this MAT gradient (Giardina et al., 2014), which appears to be related to increasing FRL within ingrowth cores observed in this study (Table 3, Model 2). Second, the negative relationship among FRL within N+P‐fertilized cores, MAT, and ${\text{NO}}_{3}^{‐}$ bioavailability suggests that the length of fine root proliferation into patches with elevated N+P is largely temperature‐dependent and bulk soil N availability ecosystem scale (Figure 1). Additionally, fine root proliferation into soil patches (*i.e.,* at the scale of the ingrowth core) with elevated N and P was significant across the gradient compared to other treatments, suggesting that at the plot scale, vegetation prioritizes localized acquisition of both N and P, but that this requirement significantly declines based on the environmental conditions of MAT on bulk soil ${\text{NO}}_{3}^{‐}$ bioavailability. The two primary findings of this study highlight the important differences between ecosystem scale (plot) and local‐scale (root ingrowth core) drivers of root morphological response to temperature and nutrient conditions, and the potential dual influence of MAT on root growth via ecosystem C fixation and via modulation of soil N bioavailability and cycling (Giardina et al., 2014; Pierre et al., 2017).

The design of this study featured conditions that should be considered in the interpretation of the root proliferation response to MAT, N bioavailability, and fertilized ingrowth cores. The one‐time fertilization treatment necessitated a shorter ingrowth duration (75 days), and therefore captured short‐term fine root responses to fertilized patches, and therefore cannot be directly compared to studies with longer ingrowth periods. The N fertilization treatment was added in the form of a urea solution and is assumed to have been converted to ammonium (${\text{NH}}_{4}^{+}$) after burial by soil microorganisms (Burton & Prosser, 2001). This may have created a contrast between the N species inside ingrowth cores and other mineral and organic N species occurring naturally in surrounding soils, which could have implications for root proliferation if plant N foraging shows discrimination among forms of N.

Co‐limitation by N and P has been observed across many terrestrial and aquatic ecosystems, where a simultaneous addition of both nutrients increases primary production above enrichments of either nutrient alone (Elser et al., 2007; Vitousek & Farrington, 1997). Observed increases in primary production in response to N+P additions reflect the stoichiometry of photosynthesis and plant growth, but these observations do not shed light on the dynamics of internal plant C partitioning for N and P acquisition. Roots respond dynamically to heterogeneous supplies of potentially growth‐limiting resources through internal signaling pathways induced by internal and external cues (Forde & Lorenzo, 2001; Hutchings & de Kroon, 1994). While diverse root responses to nutrient‐rich patches under different background nutrient conditions have been shown in model plant systems (Drew, Saker, & Ashley, 1973; Zhang & Forde, 1998), a limited number of studies have evaluated their ecological causes or consequences (Callaway, Pennings, & Richards, 2003; Chen et al., 2016; Cheng et al., 2016). Primary root initiation and elongation are linked to the immediate presence of these nutrients through root elongation gene expression (Zhang & Forde, 1998) and hormonal pathways (Drew et al., 1973; Le Deunff, Lecourt, & Malagoli, 2016). Root proliferation responses to external cues are species‐specific and seasonally dependent (Eissenstat & Caldwell, 1988; Kembel & Cahill, 2005). In the present study, these sources of variation were minimized by uniform vegetation composition, parent material, oil moisture availability across the MAT gradient and by the short‐term nature of this study. The dependence of root proliferation into N+P‐enriched patches upon both N bioavailability and MAT suggests complex interactions with environment and plant nutritional status, independent of variation in vegetation composition, soil characteristics, and water availability. Moreover, our observations illustrate the interplay between plant nutrition, fine root behavior, and environmental conditions at the stand scale (de Kroon, Visser, Huber, Mommer, & Hutchings, 2009). Because roots within ingrowth cores were not separated by species, results may not proportionately reflect the root proliferation response to N+P fertilization of all dominant plant species in the plot. If one plot‐dominant species exerts a disproportionate influence on fine root proliferation within plots, the results of this study can still be interpreted to reflect stand‐scale processes in response to MAT and N bioavailability, which may have implications for forest nutrient acquisition and associated C costs of these functions.

Proximate and ultimate limiting conditions (sensu Vitousek et al., 2010) are important to distinguish in order to characterize ecosystem responses to abiotic change. Vitousek et al. (2010) define proximate and ultimate limiting nutrients as those which influence individual biological processes and structure ecosystems, respectively. While this definition serves to contrast short versus long‐term responses to additions of different macronutrients, it should also invoke the role of abiotic conditions in determining the availability of nutrients leading to limitation (Raich et al., 1997; Vitousek & Farrington, 1997; Vitousek et al., 2010). By using an environmental gradient where environmental factors other than MAT were constrained, we were able to show the relationship between the bioavailability of an ultimate limiting nutrient, N, and an ultimate limiting condition, MAT (Pierre et al., 2017). Our results suggest that increasing N availability due to rising MAT could have consequences for fine root proliferation in soils. Decreasing fine root proliferation in N+P‐fertilized ingrowth cores with increasing MAT may indicate how tropical montane wet forests prioritize nutrient acquisition as N becomes less limiting. Unless at least one environmental condition, such as MAT, is strongly limiting, plot fertilization studies may confound the roles of first‐order variables such as temperature, pH, substrate age, and precipitation, which influence nutrient limitation of forest growth (Lambers et al., 2008; Reed et al., 2011; Vitousek, 1984).

While prior research has shown that temperature influences fine root growth and turnover (Gill & Jackson, 2000; Norby & Jackson, 2000; Pregitzer et al., 2000), and that ${\text{NO}}_{3}^{‐}$ bioavailability drives fine root production and turnover (Pregitzer et al., 1993; Robinson et al., 1999), few studies have described their interactive influences (BassiriRad, Caldwell, & Bilbrough, 1993; Leppälammi‐Kujansuu, Salemaa, Kleja, Linder, & Helmisaari, 2014; Vogt et al., 1995). In our study, MAT and N bioavailability both decrease with increasing elevation, complicating the interpretation of root foraging responses to nutrient‐enriched patches. Moreover, C availability for fine root foraging in enriched patches would be expected to increase with temperature due to concomitant increases in C cycling rates (Giardina et al., 2014; Litton et al., 2011). However, the results of our statistical model indicated that fine root length in N+P‐enriched patches increased with increasing MAT given low bulk soil N availability, while the converse was true given high bulk soil N availability (*p* = .023, Figure 1). One interpretation of this unexpected result is that root response to enriched patches is co‐dependent on C availability and background soil fertility (Robinson, 2001); in warmer, more productive climates, trees may be better able to widely explore for limiting nutrients in response to overall soil nutrient conditions (Lynch & Ho, 2005). If this interpretation is correct, it would imply that with increasing temperature and constant moisture availability, trees may be better able to overcome nutrient limitation with concomitant increases in root proliferation for nutrient acquisition as a result of overall increased productivity.

### 4.2 Mycorrhizal colonization across MAT

Through symbioses with mycorrhizal fungi, plants can compensate for soil nutrient limitation (Johnson, 2010; Rillig, 2004; Treseder, 2004) at the cost of fixed C to support the association (Hodge, 2010; Lynch & Ho, 2005). The degree of symbiosis with arbuscular mycorrhizal fungi (AMF) is a straightforward proxy for the degree of plant P limitation, though it can also reflect plant productivity (Fellbaum et al., 2012; Hawkes, Hartley, Ineson, & Fitter, 2008). We observed increasing root colonization by AMF with increasing MAT (Figure 2), which may reflect an increase in P limitation at higher N bioavailability at warmer sites (Pierre et al., 2017). Arbuscular mycorrhizae generally enhance P acquisition (Lambers et al., 2008; Rillig, 2004) and provide an advantage to plants in high‐${\text{NO}}_{3}^{‐}$ environments where P demand is typically higher (Bradley, Drijber, & Knops, 2006; Egerton‐Warburton & Allen, 2000). Increasing AMF colonization with MAT suggests that MAT could impact the ecological stoichiometry of this tropical montane wet forest by proportionally changing N bioavailability relative to P bioavailability. While we did not measure soil bioavailable P, we can infer from increasing percent AMF colonization that C allocation to mycorrhizae is increasingly prioritized at higher MAT, as plants can invest between 4% and 20% of total C budget in the AMF mutualism (Eissenstat, Graham, Syvertsen, & Drouillard, 1993; Peng et al., 1993; Watkins et al., 1996).

Colonization by AMF did not respond to any of the fertilized ingrowth core treatments, suggesting that the AMF mutualism across the gradient is driven by an interaction between productivity and bulk soil fertility, rather than the availability of nutrient‐enriched soil patches (Treseder & Allen, 2002). Plants may be able to supply more carbohydrates to support their AM symbionts at higher MAT, diverting C that would otherwise go to the root apoplast (Fitter, 2006). Our results show that AMF symbioses are more responsive to MAT than to nutrient‐rich patches, which is in contrast with our observations for fine root proliferation. Taken together, the differing patterns of fine root patch foraging and fine root AMF colonization along the MAT gradient suggest that fine roots forage in nutrient‐rich patches under N‐limited conditions at a lower C cost to the plant, while AMF are more abundant and forage more broadly under N‐rich conditions at a greater C cost to plants (Johnson et al., 1997). Increasing primary production with MAT, coupled with increasing soil N bioavailability in moist forests, may favor increased AMF colonization to maintain N and P co‐limitation.

### 4.3 Implications for ecosystem biogeochemistry

Across these tropical montane wet forest plots, a significant root proliferation response to N plus P‐enriched patches suggests that fine root foraging is most strongly influenced by the availability of both N and P. Within a stand, roots appear to be directed and elongated into patches of available N and P when background nutrient availability is low under cooler climatic conditions. The effect of background N fertility of a site appears to be temperature‐dependent in the absence of other ecosystem variation, suggesting that localized fine root growth depends on the interaction between temperature and N bioavailability. Our results suggest that increasing AM fungal colonization with MAT may be related to previously observed increasing primary production with MAT across this gradient (Giardina et al., 2014). Increasing primary production with warming may interact with temperature‐driven N availability (Pierre et al., 2017) to affect the relative C cost of mycorrhizal associations (Treseder & Allen, 2002). We hypothesize that more productive lowland tropical plant communities under warmer climatic conditions increase AMF colonization for nutrient acquisition. Further study of the dynamics of total fine root biomass and turnover across gradients of MAT paired with whole stand fertilization experiments and N availability analyses will help to determine how influential these conditions are to fine root contributions to soil C. These results add to the growing body of research demonstrating the interconnections among increasing MAT, soil nutrient availability, and plant C allocation strategies (Johnson et al. 2010; Ostonen et al., 2011; Reich et al., 2014).

## CONFLICT OF INTEREST

We declare no competing personal, financial, or institutional competing interests.

## AUTHOR CONTRIBUTIONS

**Suzanne Pierre:** Conceptualization (lead); data curation (lead); formal analysis (lead); funding acquisition (lead); investigation (lead); methodology (lead); project administration (lead); resources (lead); software (lead); supervision (lead); validation (lead); visualization (lead); writing – original draft (lead); writing – review & editing (lead). **Creighton M. Litton:** Conceptualization (supporting); methodology (supporting); resources (supporting); supervision (supporting); writing – review & editing (supporting). **Christian Giardina:** Conceptualization (supporting); resources (supporting); writing – review & editing (supporting). **Jed P. Sparks:** Conceptualization (supporting); methodology (supporting); resources (supporting). **Timothy J. Fahey:** Conceptualization (supporting); formal analysis (supporting); methodology (supporting); supervision (lead); validation (supporting); writing – original draft (supporting); writing – review & editing (supporting).

## Supporting information

Fig S1

## Data Availability

The data that support the findings of this study are openly available Dryad at https://doi.org/10.6078/D14X4B.
