# Supplementary material for: Mean annual temperature influences local fine root proliferation and arbuscular mycorrhizal colonization in a tropical wet forest
Source: Ecol Evol. 2020 Aug 28;10(18):9635–46. doi: 10.1002/ece3.6561 (PMC7520179; doi:10.1002/ece3.6561)

**Supplement 1:** Moisture content of fine roots inside fertilized fine root ingrowth cores buried in tropical montane wet forest soils in 9 permanent plots located in tropical montane wet forest on Mauna Kea, Hawaii, USA. Plots comprise a 5.2°C mean annual temperature (MAT) gradient. Fertilization treatments are nitrogen (N), phosphorus (P), combined nitrogen and phosphorus (N+P) and control with deionized water (C).


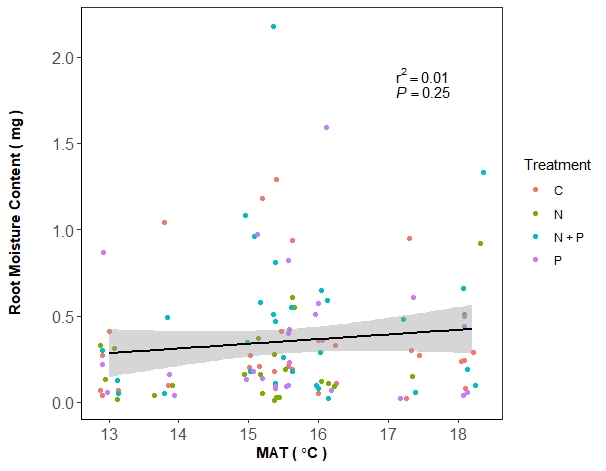

Supplement: Supplementary file 1 — Fig S1 [file ECE3-10-9635-s001.docx]
